# Heart Failure with Preserved Ejection Fraction in Women: Sex-Specific Insights Across the Continuum from Diagnosis to Outcomes

**DOI:** 10.1007/s11886-026-02392-2

**Published:** 2026-07-30

**Authors:** Sharon Andrade-Bucknor, Shiva Barforoshi, D. Elizabeth Le, Radmila Lyubarova, Jina Chung

**Affiliations:** 1 Division of Cardiology, Department of Medicine, University of Miami, 1321 NW 14th Street, Suite 510, Miami, Fl 33125 USA; 2 Division of Cardiology, Department of Medicine, Harbor UCLA Medical Center, 1000 W Carson St. Box 405, Torrance, CA 90502 USA; 3 Division of Hospital & Specialty Medicine, Section of Cardiology, VA Portland HealthCare System, Portland, OR USA; 4 Division of Cardiology, Department of Medicine, Albany Medical College, Albany, NY USA

**Keywords:** Heart failure, HFpEF, Women

## Abstract

**Purpose of Review:**

This review highlights differences in pathophysiologic mechanisms, diagnostic challenges and therapeutic responses of heart failure with preserved ejection fraction (HFpEF) in women.

**Recent Findings:**

Emerging research provides further insight into the pathophysiology of HFpEF in women, and the role of obesity and its impact on treatment. Other theories highlight the role of microvascular dysfunction in women with HFpEF. Gender differences in cardiac imaging parameters for assessment of diastolic dysfunction and elevated filling pressures may require refinement for improved diagnostic accuracy of HFpEF in women.

**Summary:**

HFpEF is fast becoming the dominant form of heart failure in the US and women have double the lifetime risk of developing HFpEF compared with heart failure with reduced ejection fraction. Gender-specific diagnostic parameters and treatment options have been hindered by low participation of women in cardiovascular trials. Increased participation in larger scale trials and further investigations into sex-specific pathophysiology are needed for improved outcomes in women.

## Introduction

Heart failure with preserved ejection fraction (HFpEF) now accounts for about 50% of all heart failure cases and is expected to become the predominant form in the US. With an aging population and increasing prevalence of comorbidities like diabetes, hypertension, and obesity, one in ten people will be affected by HFpEF over their lifetime [1]. The higher burden of comorbidities and longer life expectancy in women likely contribute to the greater prevalence of HFpEF, with a relative lifetime risk (RLR) of 10.7% - nearly twice that of heart failure with reduced ejection fraction (HFrEF), estimated at 5.8% [2]. For men, the RLR of HFpEF vs. HFrEF is similar (10.4% vs. 10.6%). More than 80% of heart failure cases in older women in the community are due to HFpEF [3]. Despite this, there remains an under-representation of women in clinical trials. Although women’s participation in HFpEF trials is higher than in HFrEF studies, it remains insufficient and warrants further improvement (Table 1).

**Table 1:** Key HFpEF trials and registries highlighting sex-specific findings and representation in medical and advanced therapies

Therapy Type	Trial / Registry	Comparator / Design	*N*	% Women	Mean Age	Sex-specific outcomes reported	Specific sex findings reported
Medical Therapy	**RELAX – 2013** [5]	Sildenafil vs. placebo	216	48%	69	No	
	**TOPCAT – 2014** [6]	Spironolactone vs. placebo	3,445	52%	69	Yes [7]	Post-hoc: potentially greater mortality reduction in women
	**PARAGON-HF – 2019** [8]	Sacubitril/Valsartan vs. valsartan	4,822	52%	73	Yes [9]	Women had greater benefit vs. men (interaction significant)
	**EMPEROR-Preserved – 2021** [10]	Empagliflozin vs. placebo	5,988	45%	72	Yes [11]	Treatment effect consistent by sex
	**DELIVER – 2022** [12]	Dapagliflozin vs. placebo	6,263	44%	72	Yes [13]	Treatment effect consistent by sex
	**PARABLE – 2023** [14]	Sacubitril/Valsartan vs. valsartan	250	38%	72	No	
	**STEP-HFpEF – 2023** [15]	Semaglutide vs. placebo	529	56%	69	Yes [16]	Similar HF benefit; women with greater weight loss
	**SUMMIT – 2023** [17]	Tirzepatide vs. placebo	731	54%	65	Yes [1, 18]	Outcomes consistent by sex
	**FINEARTS-HF – 2024** [19]	Finerenone vs. placebo	6,000	45%	72	Yes [20]	Effects consistent across sex
Advanced Therapy	**CHAMPION (Registry) – 2011** [21]	CardioMEMS vs. usual care	550	23%	62	No	
	**GUIDE-HF (Registry) – 2021** [22]	CardioMEMS vs. usual care	1,000	33%	70	Yes [23]	Effects consistent across sex
	**REDUCE LAP-HF II – 2022** [24]	Interatrial shunt vs. sham	621	62%	72	Yes [25, 26]	Women had different recurrent HF event response (interaction significant)
	**MONITOR-HF (Registry) – 2023** [27]	CardioMEMS vs. usual care	348	24%	69	Yes [28]	Sex subgroup analyzed; no major interaction

In addition to the common predisposing comorbidities, females exhibit unique, sex-specific pathophysiological mechanisms that contribute to HFpEF, with certain phenotypes more likely in females.

Furthermore, preclinical parameters for HFpEF have not been clearly defined. However, according to the Get With The Guidelines-HF registry, among those who required hospitalization, outcomes with HFpEF are similar to HFrEF with comparable 5-year mortality rates [4]. HFpEF is a major cause of morbidity and mortality, imposing a significant burden on healthcare systems.

**Fig. 1:**
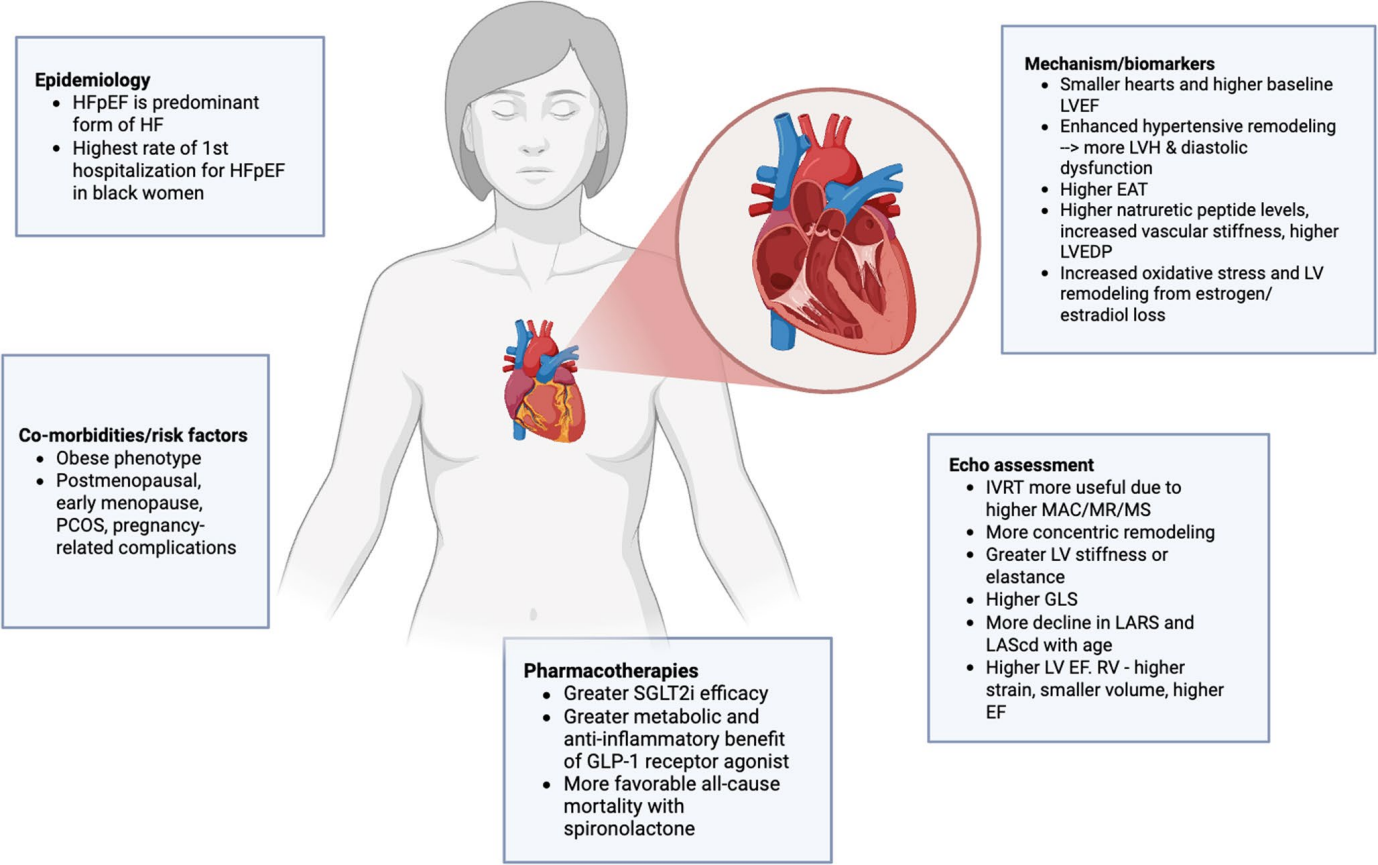
HFpEF in women: Sex-specific characteristics compared to men. *EAT* epicardial adipose tissue, *EF* ejection fraction, *GLP-1* glucagon-like peptide-1, *GLS* global longitudinal strain, *HF* heart failure, *HFpEF* heart failure with preserved ejection fraction, *IVRT* isovolumic relaxation time, *LARS* left atrial reservoir strain, *LAScd* left atrial conduit strain, *LV* left ventricle, *LVEDP* left ventricular end diastolic pressure, *LVEF* left ventricular ejection fraction, *LVH* left ventricular hypertrophy, *MAC* mitral annular calcification, *MR* mitral regurgitation, *MS* mitral stenosis, *PCOS* polycystic ovarian syndrome, *RV* right ventricle, *SGLT2i* sodium-glucose cotransporter-2 inhibitor. (Created in BioRender. Barforoshi, S. (2026) https://BioRender.com/bhf9kth.)

This review focuses on the epidemiology, pathophysiology, diagnosis, treatment, and outcomes of HFpEF in women and highlights specific areas for further research to improve their clinical outcomes. Figure 1 illustrates the sex-specific differences in these areas.

## Epidimiology & Sex-Specific Risk Factors

HFpEF prevalence is increasing overall and is especially high in women, accounting for 55–65% of heart failure cases. HFpEF is the predominant form of heart failure in older women and is associated with age-related increases in left ventricular fibrosis, reduced elasticity, and consequent diastolic dysfunction. In addition, age, ethnicity and geography influence HFpEF prevalence, reflecting differences in risk factors and healthcare access. In the US, prevalence is greatest in the South, followed by the Midwest, Northeast and least in the West [29, 30]. Black women have the highest rate of first hospitalization for HFpEF compared with Black men and White individuals [1].

Yeh et al. found that women with HFpEF were older (82.2 vs. 80.6 years; *P* = 0.029) and had higher rates of asthma, osteoarthritis, depression, hypothyroidism, and anxiety compared to men. The odds of HFpEF increased with age, BMI, rheumatological disorders, depression, anxiety, asthma, and hypothyroidism [31]. A systematic review and metanalysis of 61 studies found consistent associations between HFpEF and atrial fibrillation, diabetes, hypertension, obesity, and myocardial infarction, but results were inconclusive for chronic kidney disease [32]. Women – especially those over 60 – have a higher prevalence of hypertension and obesity than men [33], contributing to over 90% of HFpEF risk in Black women and 72% in Hispanic women [29].

The risk of developing HFpEF differs between women and men. Women are more likely to develop left ventricular hypertrophy and diastolic dysfunction in response to hypertension, and obesity poses a greater risk for heart failure in women than men [34]. Although men have a higher prevalence of diabetes, diabetic women experience a 3–7-fold greater increase in cardiovascular risk, compared to 2–3-fold in men. Women with diabetes also have a higher incidence of heart failure, though HFpEF-specific data is lacking compared to men [33].

### Obesity

Women more commonly exhibit an obese phenotype with metabolic syndrome and diabetes, whereas men more commonly have coronary artery disease. About 46% of women and 35% of men with HFpEF are obese, with more pronounced disease development in Black than in White women. Overweight and central obesity, defined by BMI ≥ 27.5 kg/m2 and waist circumference > 90 cm in females or > 100 cm in males, are correlated with HFpEF [35].

In a 10-year survey of over 9,000 adults, BMI misclassified 48% of women - who met obesity criteria by body-fat assessment (x-ray absorptiometry) and had elevated leptin levels – but not in men, potentially leading to under-recognition of obesity in women [36].

Visceral adiposity contributes to HFpEF through the adipokine hypothesis, which defines three domains: downregulated molecules (Domain I), compensatory cardioprotective factors (Domain II), and pathogenic adipokines (Domain III) [17, 37, 38]. Increased adipose tissue causes local and systemic inflammation, sodium retention, and cardiac hypertrophy and fibrosis.

Bariatric surgery increases adipokinesis in Domain I while decreasing those in Domain III. Similarly, incretin receptor agonists and established HFpEF therapies reduce visceral adiposity, suppress inflammation, improve atrial and ventricular remodeling, and enhance exercise tolerance [17, 36]. These mechanisms likely account for the findings of a sub analysis from the STEP-HFpEF trial, which demonstrated greater weight loss in women (9.6% compared to 7.2% in men), while improvement in heart failure symptoms was comparable between both sexes.

Other studies suggest pathways independent of adipokines for the observed benefits with these agents [38]. The adipokine hypothesis may be complementary to the microvascular inflammatory model also thought to play a significant role in HFpEF [39].

Epicardial Adipose Tissue (EAT), a marker of visceral adiposity, has been implicated in the pathophysiology of HFpEF [40]. Potential mechanisms include pericardial restraint that impairs ventricular filling, lipotoxicity from pro-inflammatory and pro-fibrotic cytokines, and left atrial dysfunction leading to atrial fibrillation. EAT can be measured echocardiographically, though CT and MRI provide more accurate volumetric assessments and allow evaluation of inflammation, density, and fat content [41]. Studies report mixed findings on sex differences in EAT in HFpEF, with some noting higher levels in women and others in men. Increased EAT, especially in women, is associated with greater pericardial restraint, and baseline or increasing in EAT thickness correlates with HFpEF risk [40].

### Microvascular Dysfunction

In a systematic review and metanalysis, the pooled prevalence of coronary microvascular dysfunction (CMD) in HFpEF is 71% (95% CI, 0.63–0.79). Although prevalence of CMD is similar in women and men [42], women more often show fibrosis-related biomarkers while men show inflammation-related markers [43]. CMD in HFpEF is associated with higher HF hospitalizations and cardiac death, worse diastolic dysfunction, more atrial fibrillation, and reduced strain in the left atrium (LA), left ventricle (LV), and right ventricle (RV) [43].

Studies show that low coronary flow reserve (CFR), a key feature of CMD, combined with diastolic dysfunction, increases the likelihood of developing HFpEF (hazard ratio 2.4, annualized HFpEF rate 15%). The WISE study also found low CFR to be a risk factor for HFpEF, though its prevalence was low at 3% [44].

### Estrogen, Estradiol & Pregnancy

The prevalence of HFpEF in postmenopausal women has led to significant research into the role of estrogen/estradiol (E2) loss. E2 deficiency has been linked to diastolic dysfunction through multiple genomic and nongenomic mechanisms related to increase in oxidative stress and LV remodeling [45]. Adverse effects extend beyond cardiomyocytes to fibroblasts, inflammatory cells, coronary vessels, and epicardial adipose tissue, resulting in increased inflammation, diastolic dysfunction, and LV stiffness [46]. Elevated free testosterone, as seen in the MESA study, was associated with higher LV mass and volume in women, leading to pathological remodeling [45].

In younger women, early menopause, polycystic ovary syndrome, and pregnancy-related complications (e.g., pre-eclampsia, gestational diabetes, multiparity, and recurrent pregnancy losses) are recognized risk factors for developing HFpEF [47].

## Sex-Specific Mechanims and Diagnostic Challenges

While the definition of HFpEF is widely agreed upon as left ventricular ejection fraction (LVEF) ≥ 50% with signs of congestion and elevated natriuretic peptides [30], approximately one-third of cases remain unrecognized [1]. Since women tend to have smaller hearts and a higher baseline LVEF, their ejection fraction rises more noticeably with age than in men, which may contribute to underdiagnosis [48].

HFpEF symptoms, such as dyspnea, presyncope, peripheral edema, fatigue, and weakness, overlap with many other conditions, leading to frequent misdiagnosis, especially in older women. Their symptoms are often initially attributed to respiratory disease, further complicating diagnosis [45].

### Biomarkers

Natriuretic peptides (NP) are influenced by age and obesity [1, 49], factors that are more significant in women with HFpEF. Other conditions, such as kidney disease, arrhythmias, left ventricular hypertrophy, sepsis, pulmonary embolism, and COPD, can also elevate NP and may co-exist with HFpEF. In clinical trials, NT-proBNP cutoffs have been used: >200 pg/ml or > 300 pg/ml in sinus rhythm, and > 600 pg/ml (DELIVER) [12] or > 900 pg/ml (EMPEROR-Preserved [10], PARAGON-HF [8]) in atrial fibrillation. For chronic HF in outpatient settings, a lower threshold of > 125 pg/mL is applied [57].

In healthy individuals, women have higher natriuretic peptide levels than men due to sex hormones. However, in HF, diagnostic and prognostic levels are not significantly different between sexes [50]. The PARABLE trial [14] enrolled 250 asymptomatic pre-HFpEF patients (with hypertension, diabetes, elevated BNP/NT-proBNP, left atrial volume index > 28 mL/m², LVEF > 50%), with 38.5% being women and median age of 72 years. Although blood pressure control was similar between sexes, women had higher NT-proBNP levels, increased vascular stiffness, and higher LV end-diastolic pressures, yet lower rates of hypertension, diabetes, and ischemic heart disease compared to men [14, 51].

NT-proBNP levels increase with age more in males than females, but females exhibit lower levels with increased adiposity [47]. Obesity leads to a 6–20% decrease in NT-proBNP, with a more pronounced effect in women due to increased degradation or decreased release from the myocardium [50]. As a result, older postmenopausal obese women may have normal NT-proBNP levels despite having HFpEF [47].

Additional biomarkers involved in myocardial fibrosis and/or apoptosis, such as galectin-3 (Gal-3), growth differentiation factor-15, and soluble suppression of tumorigenicity, are elevated in HF and correlate with LV end-diastolic pressure, but they do not differentiate between HFpEF and HFrEF [52]. As the list of biomarkers grows, further research is needed to assess their diagnostic and prognostic value in males vs. females, with a multi-marker approach potentially improving prognostication [53].

### Imaging

Diastolic assessment in suspected HFpEF focuses on identifying abnormal relaxation and increased filling pressures. Key to diagnosis is demonstrating elevated filling pressures at rest and/or with exercise. Echocardiography is essential for determining LVEF and assessing HFpEF mimics, such as cardiac amyloidosis, hypertrophic cardiomyopathy, pericardial disease, and ischemic heart disease. It also provides insights into regional wall motion abnormalities and significant mitral valve disease. As a primary noninvasive tool, echocardiography is validated against invasive measures for HFpEF evaluation. Cardiac MRI (CMR) offers the gold standard in volumetric data, global longitudinal strain (GLS), left atrial strain (LAS), and CMD, and can quantify intramyocardial fibrosis through extracellular volume. With preserved LVEF, women show higher extracellular volume fraction (ECV), while men have greater nonischemic late gadolinium enhancement (LGE) [54].

Recent updates to echocardiographic guidelines [55] have incorporated additional discriminators and modified some previous parameters, enhancing the accuracy of echocardiography in diagnosing HFpEF.

#### Doppler Echocardiography

The E/A ratio and mitral E deceleration time reflect LV diastolic stiffness, with a normal E/A ratio ranging from ≥ 0.8 to < 2.0. A Valsalva maneuver that decreases the E/A ratio by ≥ 50% or increases A wave velocity has a high specificity for elevated filling pressures [56]. Septal and lateral mitral annular early diastolic velocities (e’) decrease with abnormal LV relaxation and vary with age. The E/e′ ratio estimates pulmonary capillary wedge pressure (PCWP), with a lateral E/e′ ≥ 13, septal E/e′ ≥ 15, or average E/e′ ≥ 14 correlating with high filling pressures [55, 57]. No significant gender differences have been found in E, A, or e’ velocities, but E/e′ ratios were typically ± 1 point higher in women [58].

The ratio of pulmonary vein (PV) flow in systole (S) and diastole (D) depends on LV relaxation, LV and LA compliance, and LA function. PV S/D ratio of < 0.67 signifies decreased LA compliance and increased LA pressure [55]. No gender related differences have been noted in this measure though absolute S and D velocities are lower in women [59].

Isovolumic relaxation time (IVRT) is prolonged in abnormal LV relaxation with normal filling pressures but shortens as LA pressure (LAP) increases. An IVRT < 70 ms indicates elevated LAP. This measure is particularly useful in cases of significant mitral annular calcification (MAC), mitral regurgitation (MR), or mitral stenosis, which are more prevalent in older women [55, 60], where e’ and E/e’ values are not validated.

Women are more likely to develop concentric remodeling than men who have a greater likelihood of eccentric hypertrophy [61]. Greater LV stiffness or elastance occurs in women and increases more significantly with aging compared to men. More pronounced increases in LV elastance with exercise in combination with a decrease in chronotropic and contractile reserve also account for greater exercise intolerance in women [62].

The E/e’ to 3D LV end-diastolic volume ratio (DPVQ) is a non-invasive surrogate for LV stiffness that correlates well with invasive measurements. In a study of 23 HFpEF patients, higher LV stiffness was linked to elevated NT-proBNP, reduced exercise capacity, larger LAVi, thicker LV walls, and hypertension. DPVQ may enhance non-invasive HFpEF diagnosis in women, particularly during exercise.

#### Left Atrial Volume Index (LAVi)

A LAVi ≥ 34 ml/m² and elevated pulmonary artery pressures (tricuspid velocity ≥ 2.8 m/s or RV systolic pressure ≥ 35 mmHg) may indicate increased LV filling pressures. LAVi is typically ± 2 ml/m² higher in men [58]. Athletes or individuals with lone arrhythmias may have increased LAVi, and tricuspid velocities may be elevated in primary pulmonary disease. A combination of abnormal results is needed to assess diastolic function. Studies suggest that LAV indexed to height or height², rather than body surface area (BSA), provides a more accurate assessment of LA size, especially in obese patients, and may be more relevant for women with a higher incidence of obesity [63, 64].

#### Global Longitudinal Strain (GLS)

GLS decreases with age, BSA, and blood pressure [65]. While women typically have higher LV GLS than men, this is attributed to differences in BSA [66]. In a meta-analysis of 2,396 people, the mean GLS was − 21.0%, with GLS <−16% indicating significant myocardial dysfunction and 16–18% considered borderline [67–69].

Decreased GLS is seen in up to 65% of HFpEF patients (RELAX trial), suggesting subclinical LV systolic dysfunction, which correlates with biomarkers of wall stress, collagen synthesis, and diastolic dysfunction [70]. However, other studies, including a large real-world analysis, found that 18–48% of HFpEF patients had preserved GLS. These patients were more commonly female, had higher stroke volumes and arterial stiffness, and showed better outcomes [67, 71].

#### Left Atrial Strain

Left atrial strain (LAS) measures LA myocardial motion and function [72–74]. In HFpEF patients, LAS predicts major adverse cardiovascular events (MACE), all-cause and cardiovascular mortality, HF hospitalizations and atrial fibrillation development and has been used to track disease progression and treatment response [67, 73, 75]. LAS can detect elevated filling pressures earlier than other parameters like LAVi [72]. LAS has three phases: reservoir (LARS), conduit (LAScd), and booster pump/contractile (LASct). LARS reflects LA filling during ventricular systole. Women have normal LARS values ≥ 35% and abnormal values < 23%, whereas men have normal values ≥ 33% and abnormal values < 23% [76]. LARS and LAScd decline with age in both genders, but more so in women, with a less pronounced compensatory increase in LASct in women compared to men [74].

LARS, which carries the strongest prognostic value, reflects both LV systolic function and LA stiffness. Low values < 18% correlate with elevated LV filling pressures [77], and improvements in LAS are associated with reductions in filling pressures [67].

#### Right Ventricular Function

Assessing RV function in HFpEF is crucial for understanding morbidity and mortality. Impaired RV strain is defined as < 20% or < 23% with severe tricuspid regurgitation (TR) [77, 78]. RV strain is generally higher in women than men [79], though no specific parameters are used. RV dysfunction, indicated by decreasing RV free wall longitudinal strain, is present in 30–50% of HFpEF patients. RV GLS correlates with LV diastolic dysfunction and NYHA class and is an independent predictor of mortality and rehospitalization [77].

In women, RV volumes are smaller with higher RV ejection fraction (EF), and these measures correlate with clinical outcomes in HFpEF [80].

#### Pulmonary Congestion

Lung ultrasound in the emergency room can expedite the diagnosis of HFpEF by detecting multiple, diffuse, bilateral B-lines. A meta-analysis on the use of point-of-care ultrasound for diagnosing acute decompensated heart failure found a specificity of 96% and sensitivity of 78%. Adding IVC ultrasound increased specificity to 99%, but decreased sensitivity to 58% [81].

#### The Role of Exercise in Diagnosis of Diastolic Dysfunction

Since resting filling pressures are often normal in HFpEF, persistent clinical suspicion despite nondiagnostic resting diastolic parameters should prompt a diastolic stress echocardiogram [57]. This is particularly useful when impaired relaxation is present at baseline. During exercise, the E velocity increases due to impaired myocardial relaxation, and the annular e’ fails to rise appropriately, leading to a higher E/e’ ratio, reflecting increased filling pressures. An E/e’ ratio ≥ 15 is abnormal, often accompanied by a peak TR velocity > 3.4 m/s. Isolated increases in TR velocity may not reflect diastolic dysfunction, as they can occur with normal pulmonary blood flow [49].

Invasive studies show that women have poorer diastolic reserve at peak exercise, with a greater rise in PCWP and higher E/e’ ratios, indicating higher LV filling pressures [62]. A peak exercise, PCWP ≥ 20 mm Hg or PCWP/cardiac output slope > 2 mm Hg/L/min is diagnostic. Women also exhibit lower ventricular-vascular coupling, higher systemic and pulmonary vascular resistance, and lower exercise peripheral O_2_ extraction [58]. Since older women with HFpEF are typically more frail, exercise testing may be more challenging in this population.

### HFpEF Risk Calculators

Two scoring calculators have been developed to improve diagnosis of HFpEF: the H2FPEF score (Heavy, *≥* 2 antiHypertensive drugs, atrial Fibrillation, Pulmonary Hypertension, Elders aged > 60 years, and elevated Filling pressures) and the HFA-PEFF algorithm (Heart Failure Association Pre-test assessment, Echocardiography and natriuretic peptide, Functional testing, and Final etiology). The H2FPEF score was developed based on > 400 patients who underwent invasive exercise hemodynamic testing [82, 83]. Both risk calculating systems incorporate echocardiographic parameters with H2FPEF score ranging from 0 to 9 and the HFA-PEFF algorithm from 0 to 6. In the latter, a total score of ≥ 5 is considered diagnostic of HFpEF and ≤ 1 denotes a very low likelihood and would warrant evaluation for other etiologies of dyspnea [49].

In the Atherosclerosis in Communities (ARIC) study of 4,892 participants, 13.1% had unexplained dyspnea, 10.3% had known HFpEF, 76.6% were asymptomatic, and 58% were women. Tertiles of the H2FPEF and HFA-PEFF scores were assessed for rates of HF hospitalization or death over a mean follow-up of 5.3 years. Participants with unexplained dyspnea and diagnostic scores (≥ 6 for H2FPEF and ≥ 5 for HFA-PEFF) had similar risks for the primary outcome as those with known HFpEF. Both scoring systems showed that the highest probability tertile for HFpEF was more likely to be women compared to those with known HFpEF. Additionally, 28% of participants were deemed high risk by only one score, and only 4% (27 participants) were high risk by both scores [84].

Comparison of median scores of HFA-PEFF and H2FPEF showed similar values in both sexes among the TOPCAT [6] and RELAX [5] participants and RELAX and ARIC [84] patients, respectively. Median scores for HFA-PEFF differed in both sexes in the ARIC cohort and for H2FPEF in the TOPCAT cohort [35].

### Artificial Intelligence (AI) in HFpEF Diagnosis

AI applied to echocardiography can enhance HFpEF diagnosis. A 3D-Convoluted Neural Network (CNN) analyzing A4C videos achieved high accuracy and reclassified ~ 73% of indeterminate HFA-PEFF and H₂FPEF cases, with similar performance in women and men and a two-fold increase in mortality risk among AI-identified HFpEF patients [85]. In population studies (e.g., Dallas Hearts and Minds, 57% women), AI detected subclinical HFpEF and outperformed H₂FPEF [86]. External validation showed AI reduced intermediate (“non-diagnostic”) classifications compared with H2FPEF and HFA-PEFF, supporting its potential to improve diagnostic certainty in women [87].

AI-assisted phenomapping identified three HFpEF phenotypes: “older, vascular aging,” “metabolic, obese,” and “younger, natriuretic peptide deficiency.” When applied to TOPCAT (48.2% women) and RELAX (50% women) trial data, differential outcomes were observed, suggesting its potential use in clinical trials to study interventions in specific phenotypes [60].

## Treatment

### Medical Therapy

SGLT2 inhibitors significantly reduced cardiovascular events and worsening HF in HFpEF. Subgroup analyses suggested a potentially greater treatment efficacy in women compared with men. In the EMPEROR-PRESERVED trial [10], women had a 25% risk reduction vs. 19% in men, and in the DELIVER trial [12], 23% vs. 15%, though differences did not reach statistical significance. These findings may be explained by differences in LV compliance, preload sensitivity, and cardiac reserve.

In the SUMMIT trial [17] with 731 participants (53% women), the GLP-1 receptor agonist tirzepatide improved quality of life and clinical outcomes, with greater metabolic and anti-inflammatory benefits observed in women compared to men [76].

The TOPCAT trial [6], which investigated spironolactone in HFpEF patients, found all-cause mortality was more favorably impacted in females than males (HR: 0.66 vs. 1.06), with a significant sex-treatment interaction (*P* < 0.05). However, there were no sex differences in the primary endpoint of cardiovascular death, cardiac arrest, or HF hospitalizations [2].

In the PARAGON-HF trial [8], sacubitril/valsartan (ARNI) reduced the risk of first and recurrent hospitalizations by 33% in women compared to men, with no difference in cardiovascular death rates [8, 9, 88]. ARNI has a class 2b recommendation in patients with HFpEF in the 2022 AHA/ACC/HFSA guidelines [89].

Women with HFpEF may benefit from sacubitril/valsartan and spironolactone across a wider LVEF range than men because their “normal” EF can reflect greater underlying dysfunction and differential response to neurohormonal therapies.

### Advanced Therapies

In the CHAMPION [21] and MONITOR-HF [27]trials, the CardioMEMS HF System reduced recurrent HF hospitalizations and improved quality of life, although women made up a small percentage of the study populations. The GUIDE-HF trial [22], however, found no difference in outcomes.

The REDUCE LAP-HF II trial [24] on the interatrial shunting device (IASD) showed no overall difference in cardiovascular outcomes compared to the sham procedure. However, a subgroup with lower pulmonary vascular resistance (< 1.74 Woods units) saw 51% fewer HF events, with greater improvement in KCCQ-OSS scores at 24 months [26]. The RESPONDER-HF trial studying this subgroup is ongoing.

## Outcomes

In a community-based study, HFpEF patients had higher likelihood of non-cardiovascular death than those with HFrEF, and only a minority of readmissions were attributable to HF exacerbations or other cardiovascular causes. Black women have the highest rate of first hospitalization for HFpEF compared to Black men and White individuals [88].

In HFpEF, 70% of deaths in clinical trials are due to cardiovascular causes, primarily sudden death or HF. Sex-specific mortality findings are mixed, with some studies reporting higher survival in women and others showing no significant difference [45]. However, women have a higher probability of HF re-hospitalization (34% vs. 27% in men), and these re-hospitalizations are more often due to non-cardiovascular causes, with worse post-discharge outcomes. While some studies show better outcomes for women with HFpEF compared to men, the incidence of sudden cardiac death is twice as high in men [47]. Atrial fibrillation increases mortality risk in women but not in men [47, 90]Women also report worse quality of life, likely due to higher symptom burden, less social support, more depression, and greater perceived impairment [58].

Analysis from the Acute Decompensated Heart Failure Syndromes (ATTEND) registry, a multicenter cohort study in Japan, found that anemia was an independent predictor of all-cause and cardiac death in women, but not in men, with HFpEF. In contrast, anemia was an independent predictor of all-cause and cardiac death in men with HFrEF. Interestingly, anemia had no significant adverse impact on women with reduced EF [91].

In the Swedish Heart Failure registry, anemia was more prevalent in HFpEF compared to HFmrEF and HFrEF. While anemia was associated with an increased risk of death regardless of EF, it posed a greater risk of death or HF hospitalization in HFpEF and HFmrEF compared to HFrEF. This suggests that anemia may serve as a risk marker for more severe HF and cardiorenal syndrome, increasing the likelihood of adverse outcomes [92].

## Conclusion & Future Directions

The growing prevalence of HFpEF, especially in older women, along with its associated morbidity and mortality, underscores the importance of early and accurate diagnosis and increased research into targeted therapeutic options to improve outcomes. This can be achieved through the recognition of various phenotypes and their related pathophysiologic mechanisms. Novel biomarkers, along with further refinement of imaging parameters for assessing elevated filling pressures, including the use of automated software and AI, may enhance both recognition and treatment.

Future directions should include larger prospective studies to evaluate the accuracy of these measures, improve understanding of pathophysiologic differences between women and men, including the role of epicardial fat, identify preclinical markers of HFpEF that can be incorporated into clinical practice and guide sex-specific treatment strategies.

## Key References

- Yeh K, Ansar M, Jamal H, Cannon J, Thampi S, Menon R, et al. A Retrospective Comparative Study of Sex-Based Risk Variations in Heart Failure With Preserved Ejection Fraction (HFpEF) Versus Heart Failure With Reduced Ejection Fraction (HFrEF). J Prim Care Community Health 2025;16. https://doi.org/10.1177/21501319251367840.
  - ○ Findings of this study suggest greater odds of heart failure with preserved ejection fraction in women who are older with higher BMI, rheumatological or mental health disorder, asthma and hypothyroidism.
- Packer M. The Adipokine Hypothesis of Heart Failure With a Preserved Ejection Fraction: A Novel Framework to Explain Pathogenesis and Guide Treatment. J Am Coll Cardiol 2025;86:1269–373. https://doi.org/10.1016/J.JACC.2025.06.055;TAXONOMY:TAXONOMY:CME;PAGEGROUP:STRING:PUBLICATION.
  - ○ Findings of this paper attributes a central role of obesity and visceral adipose tissue to the development of heart failure with preserved ejection fraction.
- Mazur J, Gil KE, Mikrut K, Black AL, Smart S, Truong VT, et al. Exploring sex differences in myocardial fibrosis in patients with structurally normal hearts. Sci Rep 2025;15:39331. https://doi.org/10.1038/S41598-025-23059-Y.
  - ○ Findings from this study suggest that by cardiac magnetic resonance imaging, women have greater extracellular volume than men with increased ECV being associated with all-cause mortality.

## Data Availability

No datasets were generated or analysed during the current study.
